# Effects of the Donor Unit on the Formation of Hybrid Layers of Donor-Acceptor Copolymers with Silver Nanoparticles

**DOI:** 10.3390/nano13121830

**Published:** 2023-06-09

**Authors:** Věra Cimrová, Sangwon Eom, Veronika Pokorná, Youngjong Kang, Drahomír Výprachtický

**Affiliations:** 1 Institute of Macromolecular Chemistry, Czech Academy of Sciences, Heyrovského nám. 2, 162 00 Prague, Czech Republicvyprachticky@imc.cas.cz (D.V.); 2 Department of Chemistry, Hanyang University, Seoul 04763, Republic of Korea; eoms5454@gmail.com (S.E.); youngjkang@hanyang.ac.kr (Y.K.); 3 Institute of Nano Science and Technology, Hanyang University, Seoul 04763, Republic of Korea; 4 Research Institute for Natural Sciences, Hanyang University, Seoul 04763, Republic of Korea

**Keywords:** silver nanoparticles, donor-acceptor copolymers, perylenetetracarboxydiimide acceptor units, 9-(2-ethylhexyl)carbazole donor units, absorption, SEM, XPS

## Abstract

Donor-acceptor (D-A) copolymers containing perylene-3,4,9,10-tetracarboxydiimide (PDI) electron-acceptor (A) units belonging to n-type semiconductors are of interest due to their many potential applications in photonics, particularly for electron-transporting layers in all-polymeric or perovskite solar cells. Combining D-A copolymers and silver nanoparticles (Ag-NPs) can further improve material properties and device performances. Hybrid layers of D-A copolymers containing PDI units and different electron-donor (D) units (9-(2-ethylhexyl)carbazole or 9,9-dioctylfluorene) with Ag-NPs were prepared electrochemically during the reduction of pristine copolymer layers. The formation of hybrid layers with Ag-NP coverage was monitored by in-situ measurement of absorption spectra. The Ag-NP coverage of up to 41% was higher in hybrid layers made of copolymer with 9-(2-ethylhexyl)carbazole D units than in those made of copolymer with 9,9-dioctylfluorene D units. The pristine and hybrid copolymer layers were characterized by scanning electron microscopy and X-ray photoelectron spectroscopy, which proved the formation of hybrid layers with stable Ag-NPs in the metallic state with average diameters <70 nm. The influence of D units on Ag-NP diameters and coverage was revealed.

## 1. Introduction

Metal nanoparticles (NPs) such as gold (Au) and silver (Ag) nanoparticles and their composites have attracted great interest due to the localized surface plasmon resonance (LSPR) property of metal NPs exploited for many possible photonic, electronic, chemical, biological, and medical applications, such as in light-emitting devices (LEDs) including lasers [1,2,3,4], perovskite and organic solar cells [5,6,7,8,9], chemical and biological sensors [10,11,12,13,14,15], films with antibacterial, antifungal, and antiviral activity, including composite films of polyurethane with Ag-NPs with antiviral activity against SARS-CoV-2 [16,17,18,19], catalysis [20], etc. The combination of metal NPs with organic semiconductors can further improve material properties and device performance due to the LSPR effect, e.g., to increase the device efficiency.

Recently, we have prepared and characterized hybrid layers of two donor-acceptor (D-A) copolymers (CFC8-DDPDI and CFC8-EHPDI) with homogenous coverage of silver nanoparticles (Ag-NPs) with average sizes below 100 nm [21]. The CFC8-DDPDI and CFC8-EHPDI copolymers, which were used, consist of 9,9-dioctylfluorene electron-donor (D) units and *N*,*N*′-dialkylperylene-3,4,9,10-tetracarboxydiimide electron-acceptor (A) units, and they differed by the side chains attached to the perylene-3,4,9,10-tetracarboxydiimide (PDI) units (dodecyls and 2-ethylhexyls, respectively). PDI derivatives and copolymers containing this unit are of interest due to their many potential applications, namely, in photonics and electronics [22,23], such as in organic field-effect transistors and phototransistors [24,25,26,27,28,29], organic photovoltaic cells [30,31,32,33,34,35,36,37,38,39], and sensors [40,41,42,43,44,45], due to their specific physical, optical, and/or electronic properties. They represent an interesting class of n-type semiconductors [46,47] and therefore are promising as potential n-type materials for electron-transporting layers in perovskite and all-polymeric solar cells [48,49,50,51,52].

**Figure 1:**
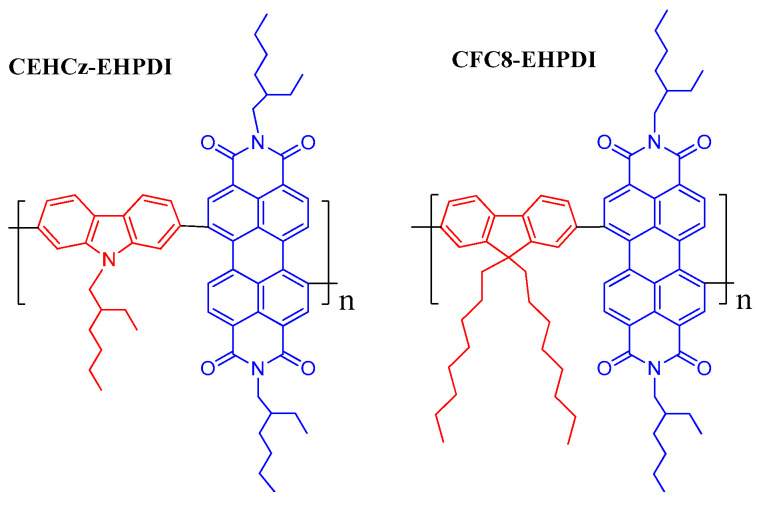
Chemical structures of the donor-acceptor (D-A) copolymers with various donor units: poly[*N*,*N*′-bis(2-ethylhexyl)perylene-3,4,9,10-tetracarboxydiimide-1,7-diyl-*alt*-9-(2-ethylhexyl)carbazole-2,7-diyl]s (CEHCz-EHPDI) and poly[*N*,*N*′-bis(2-ethylhexyl)perylene-3,4,9,10-tetracarboxydiimide-1,7-diyl-*alt*-9,9-dioctylfluorene-2,7-diyl]s (CFC8-EHPDI). The electron-donor (D) and electron-acceptor (A) units are shown in red and blue, respectively.

The CFC8-DDPDI and CFC8-EHPDI hybrid layers with Ag-NPs were prepared by electrochemical doping during the reduction of the copolymer layers using silver nitrate. We have found that the coverage and the average diameter of Ag-NPs depended on the applied potential and also on the copolymer side chains on the PDI unit. The hybrid layers exhibited significantly higher light absorption due to the LSPR property of Ag-NPs. Hence, such layers could be promising for use in solar cells. In this paper, we have studied the formation and properties of hybrid layers with Ag-NPs of the D-A copolymer CEHCz-EHPDI (Figure 1), consisting of the 9-(2-ethylhexyl)carbazole D units and the 2-ethylhexyl substituted PDI A units. The hybrid layer formation and properties are compared with those of the copolymer CFC8-EHPDI studied in our previous paper. The copolymer CEHCz-EHPDI differs from the copolymer CFC8-EHPDI by the 9-(2-ethylhexyl)carbazole D unit possessing a stronger electron-donating capability than the 9,9-dioctylfluorene D unit. The aim of this work is to investigate the effects of the D unit on the formation of Ag-NP coverage.

## 2. Materials and Methods

### 2.1. Materials

Chloroform (spectroscopic grade), acetonitrile (extra dry), tetrabutylammonium hexafluorophosphate (electrochemical grade), and silver nitrate (AgNO_3_) were purchased from commercial suppliers (Lach-Ner, Ltd., Neratovice, Czech Republic; VWR International s.r.o., Stříbrná Skalice, Czech Republic; Merck spol. s.r.o., Praha, Czech Republic; Sigma Aldrich spol. s.r.o., Praha, Czech Republic).

The copolymers CEHCz-EHPDI and CFC8-EHPDI under study were synthesized by the Suzuki coupling reaction. The synthesis and their characterization are described in our previous paper [53]. The weight-average molecular weight (*M*_w_) and dispersity (*Đ*) were for CEHCz-EHPDI *M*_w_ = 28,570, *Đ* = 2.04 and for CFC8-EHPDI *M*_w_ = 13,230, *Đ* = 1.39.

### 2.2. Layer Preparation

The pristine copolymer layers (PL) were prepared by spin-coating chloroform solution onto glass substrates covered with indium-tin oxide (ITO) in a glove box under a nitrogen atmosphere. The hybrid layers HL1 and HL2 with Ag-NP coverage were prepared by electrochemical doping of corresponding pristine copolymer layers (PL1 and PL2) during the reduction at two potentials (−1 and −1.5 V vs. Ag/Ag^+^) exceeding the potential of the first and second reduction processes for 12 min, respectively. PL1 and PL2 are pristine layers used for the preparation of hybrid layers HL1 and HL2, respectively. The numbers 1 and 2 in the abbreviations refer to the 1st and 2nd reduction processes, respectively. Thus, HL1 stands for the hybrid layer prepared by doping the pristine PL1 layer at a potential of −1 V vs. Ag/Ag^+^, corresponding to the first reduction process. HL2 means a hybrid layer prepared by doping the pristine PL2 layer at a potential of −1.5 V vs. Ag/Ag^+^ exceeding the potential of the 2nd reduction process. These abbreviations are generally used for both copolymers, along with the copolymer abbreviation for distinction. A homemade cuvette three-electrode cell with platinum (Pt) wire as a counter electrode and a non-aqueous Ag/Ag^+^ reference electrode (Ag in 0.1 M AgNO_3_ solution) connected to a PA4 polarographic analyzer (Laboratory Instruments, Prague, Czech Republic) was used. A solution of silver nitride in an electrolyte (0.1 M tetrabutylammonium hexafluorophosphate in anhydrous acetonitrile) with a concentration of 7 × 10^−4^ M was used for the hybrid layer formation. Layer thicknesses (in the range of 100–160 nm) were measured using a KLA-Tencor P-10 profilometer (KLA-Tencor Corporation, Milpitas, CA, USA).

### 2.3. Methods

UV-vis absorption spectra were recorded by a Perkin-Elmer Lambda 35 UV/VIS spectrometer (PerkinElmer Instruments, Shelton, WA, USA). They were measured in situ using the homemade cuvette described above, placed in the glove box, and connected to the UV/VIS spectrometer using fiber optics. Scanning electron microscopy (SEM) measurements were performed using a high-resolution FE-SEM (JEOL Ltd., Tokyo, Japan) JSM-7800F Prime equipped with an in-lens Schottky plus field emission electron gun and an EDS detector (resolution: 0.7 nm at 15 kV). A thin Pt conductive layer (~20 Å) was deposited on the layer surfaces before SEM measurements. A K-Alpha^+^ XPS spectrometer (ThermoFisher Scientific, Swindon, UK) operating at a base pressure of 1.0 × 10^−7^ Pa was used for X-ray photoelectron spectroscopy (XPS) measurements. The samples were analyzed using microfocused monochromatic Al Kα X-ray radiation of 72 W with spot sizes of 30 and 400 μm. Survey and high-resultion (HR) spectra were measured with a pass energy of 200 and 50 eV, respectively. The X-ray incidence angle was 30°, and the emission angle was along the surface normal. The calibration of the binding energy (BE) scale of the XPS spectrometer was performed based on well-known peak positions of polyethylene terephthalate (C 1s C–C and C–H; C–O and C(=O)–O), Cu (Cu 2p), Ag (Ag 3d), and Au (Au 4f) metals. The instrument software Thermo Avantage version 5.99.22 was used for the data acquisition and the conversion of the data files to the AVG format. The AVG datafile format was converted to the VAMAS format using CasaXPS software version 2.3.25, which was used for XPS spectra analysis [54]. The quantification was performed applying the analyzer transmission function, Scofield sensitivity factors, effective attenuation lengths (EALs) for photoelectrons, and considering the sum of C, N, O, and Ag atoms as 100% with regions up to approximately 30 eV below the peak kinetic energy. The Universal Tougaard background was used for the analysis [55].

## 3. Results and Discussion

### 3.1. Absorption Properties

**Figure 2:**
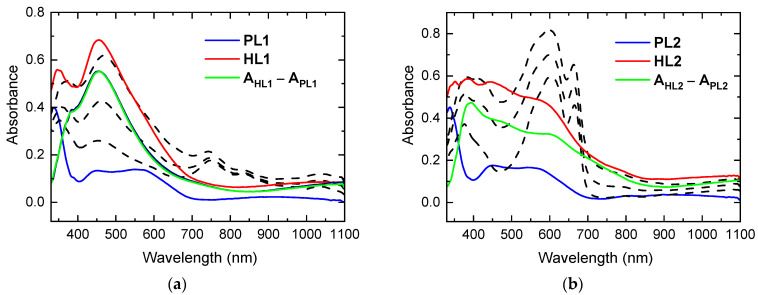
Absorption spectra of CEHCz-EHPDI pristine (PL1, PL2—blue) layers and hybrid (HL1, HL2—red) layers prepared at (**a**) –1 V (HL1) and (**b**) –1.5 V (HL2) vs. Ag/Ag^+^ corresponding to the first and second reduction processes, respectively. Absorption spectra as measured during Ag doping are also displayed (black dashed). Difference spectra (difference in the absorbance (A) of hybrid A_HL_ and pristine A_PL_ layers: A_dif_ = A_HL_ − A_PL_) are displayed by green curves.

**Figure 3:**
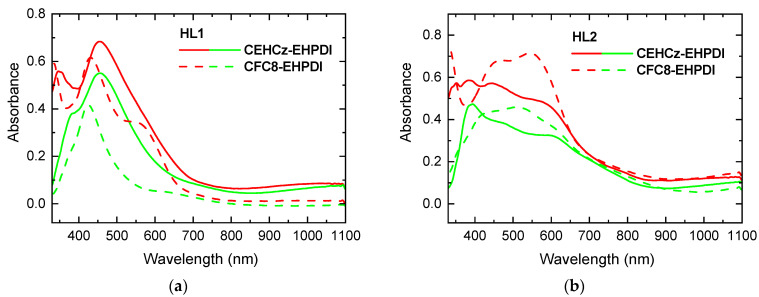
Comparison of absorption (red) and difference (green) spectra for HL1 and HL2 layers made of CEHCz-EHPDI (solid) and CFC8-EHPDI (dashed) prepared at potentials corresponding to the (**a**) first (HL1) and (**b**) second (HL2) reduction processes. The difference spectrum is the difference in the absorbance (A) of hybrid A_HL_ and pristine A_PL_ layers: A_dif_ = A_HL_ − A_PL_.

The absorption spectra of the CEHCz-EHPDI copolymer pristine layers (PL1, PL2—blue curves) and corresponding hybrid layers with Ag-NPs (HL1, HL2—red curves) prepared at two potentials, –1 V (HL1) and –1.5 V (HL2) vs. Ag/Ag^+^, i.e., when the potential reached or exceeded the first and second reduction peak potentials, respectively, are displayed in Figure 2. The numbers 1 and 2 in the abbreviations indicate the first and second reduction processes in the preparation of hybrid layers, respectively. PL1 and PL2 are pristine layers used for the preparation of hybrid layers HL1 and HL2, respectively. We use these abbreviations generally for both copolymers for distinction with reference to the respective copolymer. Absorption spectra during Ag doping were also recorded in situ and are displayed as dashed curves in Figure 2. In these spectra, the NIR bands with maxima at ca. 750 and 1030 nm correspond to the first reduction process (PDI^−^ anion), and the absorption in the visible region with two maxima at ca. 600 and 660 nm is characteristic for the second reduction (PDI^2−^ anion). These bands disappeared after turning off the potential, while a new broad absorption in the visible region with maxima located between 380 and 500 nm remained. This increase in absorption reflects changes caused by the formation of Ag-NPs and is related to the LSPR effect, for which an absorption band is characteristic in this spectral region [56,57,58]. The shapes of the absorption spectra of hybrid HL1 and HL2 layers prepared at first and second reduction, respectively, significantly differ. The absorption spectra of the HL2 layers are broader than those of the HL1 layers, and their maxima are blue-shifted. As the position of the LSPR absorption band maximum depends on the size and shape of the Ag-NPs [59], this indicates that the Ag-NP properties and coverage in HL1 and HL2 layers differ. The absorption spectra of the HL1 and HL2 layers of the CFC8-EHPDI copolymer with the 9,9-dioctylfluorene D unit also differed, but in another way, as described in detail in our previous paper [21]. The absorption and difference spectra of both copolymer hybrid layers are displayed in Figure 3 for comparison. The absorption maxima and the maxima of the difference spectra are summarized in Table 1. For the CEHCz-EHPDI copolymer with the 9-(2-ethylhexyl)carbazole D unit, the absorption spectrum of HL1 is red-shifted, whereas the HL2 spectrum is blue-shifted when compared with the corresponding spectra of HL1 and HL2 layers of the CFC8-EHPDI copolymer with the 9,9-dioctylfluorene D unit. This indicates that the D unit influences the Ag-NP formation during the first and second reduction processes. The red shift and broadening of LSPR absorption can be associated with the increased aggregation of Ag-NPs or different shapes [60,61,62,63,64]. The absorption broadening observed in the spectra of HL2 layers for both copolymers is attributed to the longitudinal SPR due to the higher amount of Ag-NP aggregation and/or formation of Ag-NP chain-like structures at higher Ag-NP coverages.

### 3.2. Scanning Electron Microscopy

**Figure 4:**
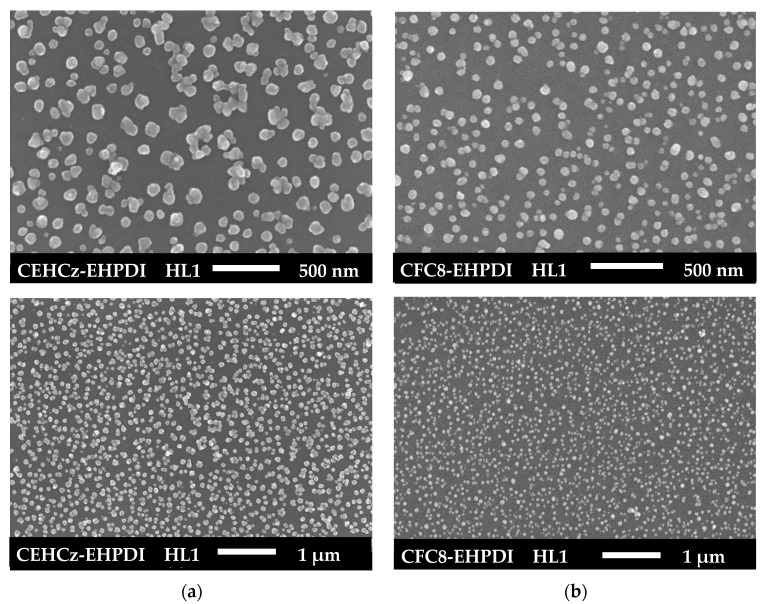
SEM images of HL1 hybrid layers of (**a**) CEHCz-EHPDI and (**b**) CFC8-EHPDI copolymers prepared at the first reduction process.

**Figure 5:**
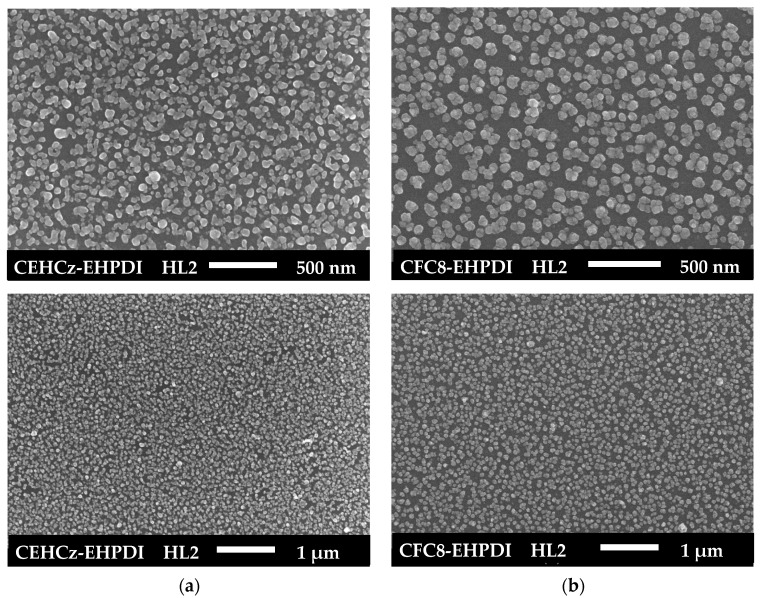
SEM images of HL2 hybrid layers of (**a**) CEHCz-EHPDI and (**b**) CFC8-EHPDI copolymers prepared at the second reduction process.

The surface morphologies of hybrid layers were studied by SEM to prove the presence of Ag-NPs and to determine their size and distribution. SEM images of pristine PL and hybrid HL1 and HL2 layers made of copolymers, which differ by the donor units, are shown in Figure 4 and Figure 5. The Ag-NP surface coverage and diameters were evaluated from the SEM images using ImageJ 1.53t software. Differences in both coverage and diameters were found for the copolymers with different D units. The results of the analysis are summarized in Table 1. Higher Ag-NP surface coverage values were evaluated for the hybrid layers made of the CEHCz-EHPDI copolymer with the 9-(2-ethylhexyl)carbazole D unit, possessing the stronger D ability, than for the layers made of the CFC8-EHPDI copolymer with the 9,9-dioctylfluorene D unit. For both copolymers, the coverage is higher for the HL2 layers prepared at the potential exceeding the second reduction process. It reached 41% for the CEHCz-EHPDI HL2 layer and 39% for the CFC8-EHPDI HL2 layer (see Table 1). A more pronounced difference in the Ag-NP surface coverage was found for the HL1 layers prepared at the first reduction process. In this case, the Ag-NP surface coverage values of 27 and 20% were evaluated for the HL1 layers of CEHCz-EHPDI and CFC8-EHPDI, respectively.

**Figure 6:**
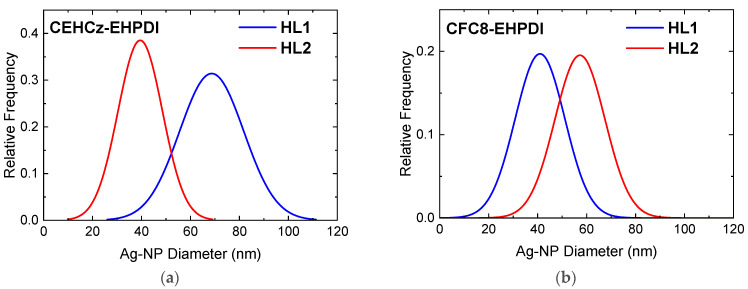
Normal distribution functions of the Ag-NP diameters evaluated from SEM images of hybrid layers of (**a**) CEHCz-EHPDI and (**b**) CFC8-EHPDI copolymers prepared at the first (HL1) and second (HL2) reduction processes.

The Ag-NP sizes (diameters) differ for the HL1 and HL2 layers, i.e., layers prepared under different conditions, and also for the copolymers under study. For CEHCz-EHPDI hybrid layers, larger diameters are observed for the HL1 layers than for the HL2 layers. This is the opposite result when compared with the CFC8-EHPDI hybrid layers, where Ag-NPs with larger diameters are formed in the HL2 layers than in the HL1 layers. The SEM analysis using Image J provided histograms of the Ag-NP diameters, which were fitted with the normal distribution function (Gaussian) given by the general formula for the probability function *f*(*x*) expressed:

*f*(*x*) = *f*_0_ + *A* exp(−(*x* − *d*_0_)^2^/2*σ*^2^) (1)

where *d*_0_ is the mean diameter and σ is the distribution parameter. The evaluated parameters *d*_0_ and σ are given in Table 1. The normal distribution functions are displayed in Figure 6, where the comparison for the copolymers CEHCz-EHPDI with 9-(2-ethylhexyl)carbazole and CFC8-EHPDI with 9,9-dioctylfluorene D unit is shown. These SEM results correlate well with the absorption spectra. They explain the above-mentioned red shifts of the HL1 absorption spectra and the blue shifts of the HL2 absorption spectra of CEHCz-EHPDI copolymer compared to the corresponding HL1 and HL2 absorption spectra of CFC8-EHPDI copolymer. An aggregation of Ag-NPs into chain-like structures appeared in HL2 layers with high surface coverage, which is in accord with the broad HL2 absorption spectra. These results are consistent with surface plasmon theory [56].

### 3.3. X-ray Photoelectron Spectroscopy

**Figure 7:**
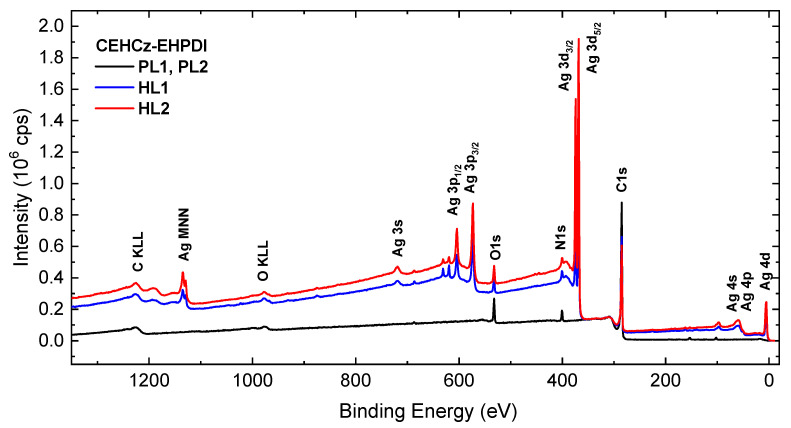
XPS-wide spectra of the pristine (PL-black) and hybrid layers of CEHCz-EHPDI copolymer prepared at the first (HL1-blue) and second (HL2-red) reduction processes.

XPS was used particularly to identify the state of the Ag-NPs. The XPS wide (survey) and high-resolution (HR) spectra were measured for both the PL and HL layers. An example of the XPS-wide spectra of the PL and HL layers for the CEHCz-EHPDI copolymer is displayed in Figure 7.

XPS-wide spectra of PL layers contain signals from C, O, and N elements of the copolymers and the spectra of HL layers in addition to Ag characteristic peaks, which can be assigned to Ag 4d, Ag 4p, Ag 4s, Ag 3d_5/2_, Ag 3d_3/2_, Ag 3p_3/2_, Ag 3p_1/2,_ and Ag 3s photoelectrons and Ag MNN Auger lines [65]. With the increasing Ag-NP coverage, the inelastic background also increased due to inelastically scattered photoelectrons [66]. To prove the metallic state of Ag-NPs, Auger parameters (APs), α_4_ and α_5_, were calculated according to the relation:

α_4_ (α_5_) = BE_Ag3d_ + KE_4_ (KE_5_) (2)

where BE_Ag3d_ is the experimental binding energy (BE) of the Ag 3d_5/2_ component maximum and KE_4_ and KE_5_ are the kinetic energies of the two most intense peaks M_4_N_45_N_45_ and M_5_N_45_N_45_ in the Auger electron structure, respectively. For all hybrid layers, the evaluated α_4_ and α_5_ values were 725.9–726.0 eV and 720.3–720.5 eV, respectively. These values are in good agreement with those for Ag in the metallic state, which are nearly 2 eV higher than the values for any other oxidized Ag species or Ag in other chemical states [67]. As an example, the lower α_4_ and α_5_ values of 723 and 718.2 eV, respectively, were evaluated for AgNO_3_. Hence, we can rule out any chemical reaction between Ag and the O or N atoms of the copolymer. A partial oxidation of Ag due to the interaction with N atoms was reported, for example, for the Ag-NP-conducting polymer polyaniline [68].

**Figure 8:**
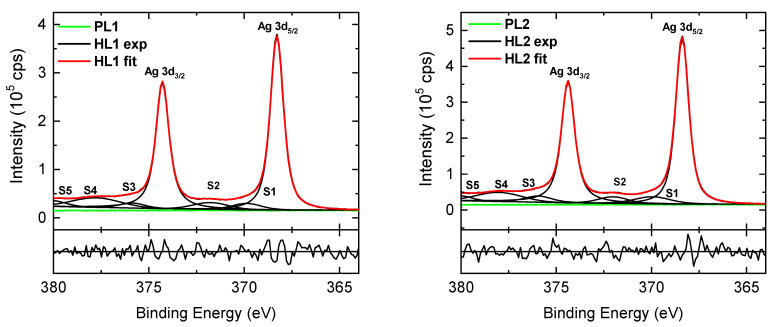
High-resolution Ag 3d spectra of CEHCz-EHPDI pristine (PL1, PL2) and hybrid (HL1, HL2) layer surfaces, including components. Normalized residuals are shown below the spectra.

The Ag-NP metallic state was further confirmed by analysis of the HR spectra. The modeling of HR C 1s, Ag 3d, N 1s, and O 1s spectra was performed using the Voigt profile. The residual standard deviation value, which was <1.17, was used to check the goodness of fit. Normalized residuals are displayed below the spectra. The fit goodness was also indicated by the residuals to the fit and Monte Carlo-based error analysis. An example of HR Ag 3d spectra for CEHCz-EHPDI layer surfaces is shown in Figure 8. The characteristic peaks correspond to Ag 3d_5/2_–Ag 3d_3/2_ spin-orbit components. The peak separation of 6 eV is in good agreement with the spin-orbit splitting separation value for metallic Ag [69]. In the Ag 3d spectra of the corresponding PL layers, no peaks indicating any Ag presence were found. The Ag 3d_5/2_ peak positions (BEs) in the Ag 3d spectra of the hybrid layers are in good agreement or slightly up-shifted (<0.1 eV) compared with the Ag 3d_5/2_ BE value of 368.28 eV for the metallic Ag. The most pronounced up-shifts were observed for the HL2 layers with the highest coverages and could be explained by charge redistribution at the surface and interface [70]. The explanation of the slight BE up-shifts was also supported by the AP values. The HR Ag 3d spectra were deconvoluted into two Ag 3d_5/2_ and Ag 3d_3/2_ main components and additional symmetrical lines corresponding to the satellites analogously to the metallic Ag spectrum. The HR Ag 3d spectra of CEHCz-EHPDI hybrid layer surfaces exhibited similar features as the HR Ag 3d spectra of CFC8-EHPDI hybrid layer surfaces, which are analyzed in detail in our previous work [21]. The satellites S1 and S3, which are separated by ca. 1.5 eV relative to the main Ag 3d_5/2_ and Ag 3d_3/2_ peaks, respectively, can be interpreted as an emission process associated with an atomic-like pure p-screening. The S2 and S4 satellites, separated by ca. 3.5 eV relative to Ag 3d_5/2_ and Ag 3d_3/2_ BEs, respectively, are associated with the plasmon loss features. The S5 satellite separated ca. 12 eV from Ag 3d_5/2_ BE, which corresponds to the 4d → 5p shake-up [71,72,73].

**Figure 9:**
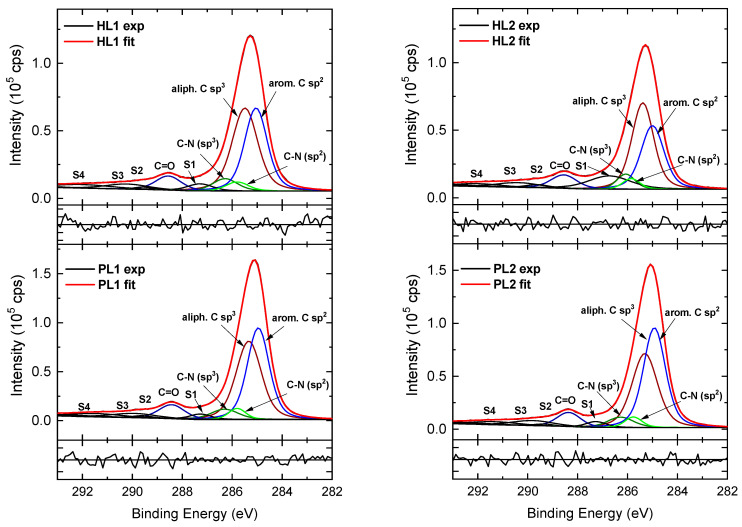
High-resolution C 1s spectra of CEHCz-EHPDI pristine (PL1, PL2) and hybrid (HL1, HL2) layer surfaces, including components. Normalized residuals are shown below the spectra.

**Figure 10:**
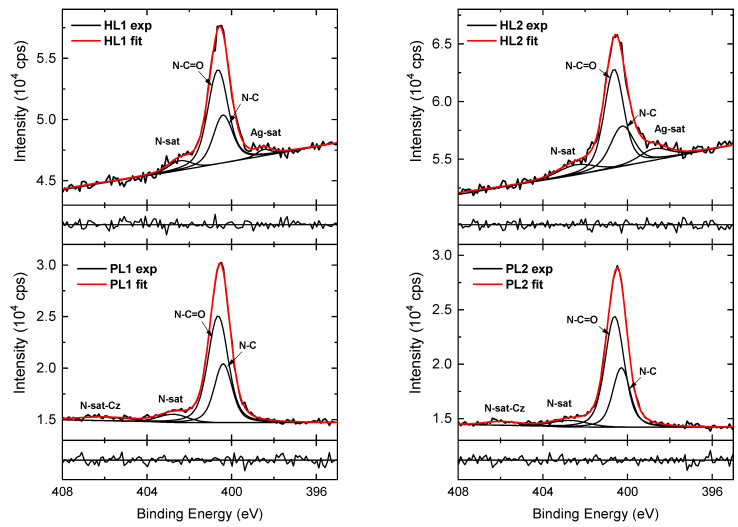
High-resolution N 1s spectra of CEHCz-EHPDI pristine (PL1, PL2) and hybrid (HL1, HL2) layer surfaces, including components. Normalized residuals are shown below the spectra.

**Figure 11:**
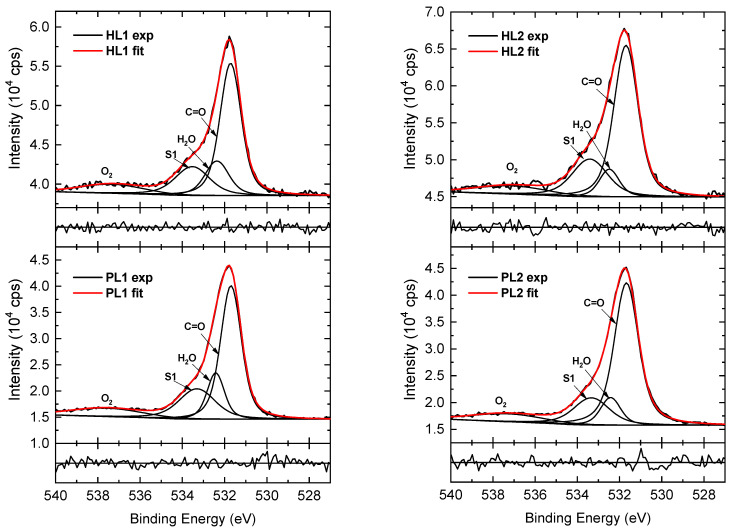
High-resolution O 1s spectra of CEHCz-EHPDI pristine (PL1, PL2) and hybrid (HL1, HL2) layer surfaces, including components. Normalized residuals are shown below the spectra.

The HR C 1s, N 1s, and O 1s spectra of PL and HL layer surfaces for CEHCz-EHPDI are shown in Figure 9, Figure 10 and Figure 11. The differences in modeling the spectra of the CEHCz-EHPDI and CFC8-EHPDI layers are in the deconvolution of the C 1s and N 1s spectra due to the difference in the D unit. The set of chemically shifted components with different BEs corresponding to the different chemical states of carbons increased in HR C 1s spectrum analysis due to the different environment of the N atom in the carbazole unit compared to the fluorene unit. In the modeling of the C 1s core-level spectra of the pristine layers, the component areas were constrained to correspond to the chemical structure of the copolymer repeat units.

The HR C 1s spectra of the CEHCz-EHPDI layers, which are displayed in Figure 9, were deconvoluted into 5 components corresponding to aromatic C sp^2^, aliphatic C sp^3^, C–N sp^2^, C–N sp^3^, and C=O groups, and π-π* satellite components, which typically appear in the XPS spectra of aromatic compounds. In the HR spectra of the PL layers, the component peak maxima are located at 284.9 eV (aromatic C sp^2^), 285.3 eV (aliphatic C sp^3^), 285.8 eV (C–N sp^2^), 286.3 eV (C–N sp^3^), and 288.4 eV (O=C–N), which is in good agreement with the data reported in the literature [74,75,76]. The peak assigned to the shake-up excitations associated with the aromatic C sp^2^ is located at approximately 287.3 eV, which also agrees well with that observed in the spectra of PDI derivatives [77,78]. The other weaker peaks at higher BEs are associated with shake-up phenomena—π-π* satellite peaks of C=O and aromatic C sp^2^ [76,77,78,79]. A decrease in the main peak area and an increase in the satellite/main peak area ratios are observed in the spectra of the hybrid layers, similar to the HR C 1s spectra of CFC8-EHPDI HL layers as analyzed in detail in our previous work [21].

The HR N 1s spectra of the PL layers of the CEHCz-EHPDI copolymer are displayed in Figure 10. They have to be modeled with four components. Compared to modeling the CFC8-EHPDI spectra, two additional components are required due to the N atom in the carbazole unit. The components with maxima at 400.3 and 400.6 eV are assigned to N–C in carbazole D units and N–C=O in PDI A units, respectively [76]. The smaller peaks at higher BE of approximately 403 and 406 eV represent π-π* satellite features of PDI and carbazole units, respectively, which are characteristic of nitrogen-containing aromatic polymers. In the HR N 1s spectra of the hybrid layers, the components corresponding to the C–N and N–C=O groups also dominate, but the intensity of both peaks decreased compared with that in the spectra of the corresponding PL layers. An additional component at a lower BE in the N 1s energy region, which is influenced by the Ag 3d photoelectron losses, can be assigned to the Ag 3d core line satellite (bulk plasmon) [71]. The contribution of the satellite component increases with increasing Ag 3d doublet peaks, i.e., it is higher in HL2 layers with higher Ag-NP coverages than in HL1 layers.

The HR O 1s spectra of the CEHCz-EHPDI layers displayed in Figure 11 were deconvoluted into four components. The main contribution, with a maximum at 531.68 eV, is assigned to the C=O of the imide groups in the copolymer PDI unit, and the peak at 533.3 eV to the shake-up [80]. The other two components originate from air contamination by adsorbed water (maximum at ca. 532.4 eV) and oxygen (maximum at ca. 537.3 eV) [81,82,83,84]. The air contamination content differs for the PL and corresponding HL layers. It is higher for the PL1 than PL2 layers, as is evident from the comparison of the corresponding component peak areas. The relative area of the peak corresponding to the adsorbed water is higher in the PL1 spectrum than that in the PL2 spectrum, which is consistent with the quantitative analysis, where a higher amount of oxygen was evaluated for PL1 than for PL2. In the HR O 1s spectra of the hybrid layers, the main peak area assigned to the C=O of the imide groups and the adsorbed H_2_O peak area decreased, while an increase in the satellite/main peak area ratio is observed. The detailed analysis of the HR spectra of the CFC8-EHPDI PL and HL spectra given in our previous paper also revealed differences in the air contamination contributions, but the main features exhibit the same character.

The atomic surface Ag concentration using traditional XPS quantification analysis gave values of 15 and 23% for CEHCz-EHPDI HL1 and HL2 layers, respectively, which are higher values than those for CFC8-EHPDI HL1 (12%) and HL2 (19%) layers. The results correlate well with the results of the Ag-NP coverage values.

## 4. Conclusions

The effects of the D unit on the formation of Ag-NPs on CEHCz-EHPDI and CFC8-EHPDI copolymer layers during reduction processes were studied. The absorption spectra of the hybrid layers of the CEHCz-EHPDI with the 9-(2-ethylhexyl)carbazole D units and the CFC8-EHPDI with the 9,9-dioctylfluorene D units differ. The differences in absorption agree with the differences in the Ag-NP diameters and coverages evaluated from the SEM images. For the copolymer CEHCz-EHPDI with the 9-(2-ethylhexyl)carbazole D units possessing the stronger donor character, larger diameters were evaluated for the HL1 layers prepared at the first reduction process than for the HL2 layers prepared at the second reduction compared to the copolymer CFC8-EHPDI with the 9,9-dioctylfluorene D units, where larger diameters were determined for the HL2 layers prepared at potentials corresponding to the second reduction than those for the HL1 layers prepared at the first reduction process. Hybrid layers made of CEHCz-EHPDI exhibited higher Ag-NP surface coverages, corresponding to higher atomic Ag concentrations, than hybrid layers made of CFC8-EHPDI. Analysis of the wide and HR XPS spectra proved the metallic state of the Ag-NPs. The hybrid layers with various Ag-NP coverages up to 41% exhibited significantly higher light absorption than pristine layers due to the plasmonic effects of Ag-NPs.

## Figures and Tables

**Table 1:** Absorption maxima of hybrid (HL1, HL2) layers (*λ*max), maxima of difference spectra (*λ*_difmax_), Ag-NP coverage (*A*_Ag-NP_), and parameters of the normal distribution (*d*_0_, *σ*). The main maxima are printed in bold.

Copolymer	Layer	*λ*_max_ (nm)	*λ*_difmax_ (nm)	*A*_Ag-NP_ (%)	*d*_0_ (nm)	*σ* (nm)
CEHCz-EHPDI	HL1	347, **455**	456	27	68.7	13.1
	HL2	352, **384**, 445	394	41	41.4	11.6
CFC8-EHPDI	HL1	335, **431**	425	20	41.0	10.1
	HL2	336, 472, **546**	514	39	57.3	10.2

## Data Availability

Not applicable.
